# Research on the bearing creep characteristics and constitutive model of gangue filling body

**DOI:** 10.1038/s41598-024-66271-y

**Published:** 2024-07-02

**Authors:** Pengfei Wu, Bowen Chen, Bing Liang, Weiji Sun, Jiaxu Jin, Zhiqiang Lv, Jihe Zhao, Zhenbo Gao

**Affiliations:** 1 School of Civil Engineering, Liaoning Technical University, Fuxin, 123000 Liaoning China; 2 School of Mechanics and Engineering, Liaoning Technical University, Fuxin, 123000 China

**Keywords:** Deep filling, Three-directional compaction, Gangue, Creep test, Constitutive model, Engineering, Environmental impact

## Abstract

The creep characteristics and potential deformation patterns of gangue backfill material are crucial in backfill mining operations. This study utilizes crushed gangue from the Gangue Yard in Fuxin City as the research material. An in-house designed, large-scale, triaxial gangue compaction test system was used. Triaxial compaction creep tests were conducted on gangue materials with varying particle size distributions. Analysis was performed based on different particle sizes, stresses, and confinement pressures. The study investigates the creep characteristics of the gangue under different conditions and explores the underlying causes. It reveals the relationship between the creep deformation of gangue materials and the passage of time. Mathematical methods are applied to develop a triaxial compaction creep power law model for gangue backfill materials. Finally, the creep results are fitted using an empirical formula approach.

## Introduction

Coal plays a central role as an essential resource for the development of society. However, a significant amount of solid waste is generated during the manufacturing process. This also triggers various environmental issues. For example, after coal has been mined, the overburden forms mountains^1–3^. At the same time, the dust generated during the accumulation process of gangue mountains, together with spontaneous combustion, has an impact on people's living environment^4^. Not only does this result in a significant waste of land resources, but it also further degrades the ecological environment of the mining area. Backfill mining technology is an important technical approach in line with the strategy of sustainable development. It can improve the utilization efficiency of coal resources. At present, the intelligent sorting of the underground gangue is in progress. In-situ backfill of deep mining areas. Establishment of a comprehensive system of deep well backfilling technology. Achieve a high utilization and recovery gangue mining method^5,6^. Mechanized solid backfill coal mining technology. Direct non-cemented dense backfilling of solid waste in the mining area. Has achieved favorable economic benefits and technological innovation^7–9^. The development of backfill mining technology using discarded gangue as backfill. It can effectively solve environmental problems such as gangue pollution^10–12^. However, the surrounding rock is disturbed after excavating deep coal resources. The environmental conditions change significantly after gangue filling, inevitably altering the mechanical properties and long-term performance of the gangue backfill body^13,14^. Therefore, the study of the compaction and creep mechanical properties of gangue fills is of great importance for safe and efficient mining in the mining area.

Many researchers are currently investigating the compaction and creep properties of gangue backfill material. Guo et al.^15,16^ selected two types of gangues for compression testing and described the stress–strain relationship using a logarithmic function. Based on the Singh-Mitchell creep model, a stress–strain-time creep model for gangue has been established, providing a reference for the design of gangue backfill mining. Sun et al.^17–19^ developed a creep testing apparatus for geopolymer cemented coal gangue-fly ash backfill and incorporated the fractional order Burger's model. The creep disturbance law was investigated, and a creep disturbance constitutive model for geopolymer gangue backfill was established. Hou et al.^20^ considered the influence of seepage-stress coupling effect on the long-term stability of gangue backfill, revealing the deformation characteristics of backfill under coupling effects, establishing a creep constitutive model, and specifying the impact of stress levels on creep parameters. This research provides crucial experimental evidence for the safety of underground coal mine backfill mining. Huang et al.^21^ used a large-scale compression testing apparatus to analyze the viscoelastic properties of gangue. Based on a fractional viscoelastic foundation model, constructed a high-precision graded viscoelastic creep model. The variation law of the control effect of the filling material's viscoelasticity on the roof was obtained. This provides theoretical references for the requirements of filling materials in mine filling and long-term dynamic deformation prediction. Wu et al.^22^ investigated the creep and mechanical properties of cemented waste rock backfill (CWRB). They used the Burgers model to characterize the creep behavior of the backfill material, validated the creep model using a genetic algorithm, and investigated the effect of the creep behavior of CWRB on the structural rock. Chen et al.^23,24^ performed uniaxial compression and creep tests on backfill paste and analyzed the variations in physical properties and other parameters of the backfill material. They elucidated the macroscopic hardening mechanism of the backfill under creep conditions. Zhou et al.^25^ conducted creep tests and introduced a damage variable to describe the degradation pattern during the creep stages. They developed a creep damage model for fully saturated backfill material that considers both time and stress.

On the other hand, there is an inseparable relationship between the compaction deformation characteristics and the creep properties of gangue backfill materials. Zhang et al.^26^ investigated the relationship between the deformation of crushed gangue and the stress level and gradation characteristics through lateral compression tests. The research findings indicate that the stress of crushed gangue exhibits exponential growth with increasing strain, and there is a positive correlation between the compressive modulus and the compression rate. Liu et al.^27^ conducted laboratory experiments and numerical simulations to investigate the bearing characteristics and compression deformation patterns of gangue backfill materials with varying particle sizes. They found that as the particle size of gangue increases, the gangue particles are more susceptible to experiencing force chain instability and failure. Dong et al.^28,29^ proposed using both washed gangue and crushed gangue as solid filling materials. They employed a similar gradation method to prepare test samples and conducted combined compression tests to compare and analyze compression characteristics. The results indicate that the mixed gangue enhances compressive performance and greatly promotes the consumption of waste gangue. He et al.^30^ utilized self-designed steel cylinders and a hydraulic press to conduct compaction tests on gangue backfill with different particle sizes and loading rates. They analyzed post-crushing stress–strain characteristics, energy dissipation, and other properties, concluding that the particle gradation of gangue has a more significant impact on its compaction characteristics. Huang et al.^31^ investigated the influence of particle size distribution and confining pressure conditions on the deformation and failure characteristics of gangue solid waste. Through triaxial compression tests, they revealed the impact of particle breakage on gangue solid waste and obtained the strain characteristics of gangue solid waste under compressive loading. Wu et al.^32^ conducted an in-depth investigation into the compression characteristics of mixed-size gangue backfill materials based on the Talbot theory. This team employed the discrete element numerical simulation method to study the fragmentation mechanism of mixed-size gangue particles. However, there is limited research on the mechanical bearing properties of gangue in different gradations for different conditions. Therefore, it is necessary to investigate the mechanical properties of gangue and analyze its working mechanism.

The above research results mainly investigated the influence of stress conditions on the creep properties of backfill materials and the mechanical properties of the backfill body under compressive crushing action. However, there is limited research on the compressive crushing load-bearing characteristics between differently graded gangue materials and the creep characteristics concerning loading stress and time. Therefore, this study uses a proprietary large-scale gangue triaxial compaction test system to investigate the creep characteristics of different graded gangue backfill materials. The creep characteristics and developmental patterns of gangue filling materials were analyzed from various perspectives. Using a power law expression, establish a triaxial compaction creep power law model for gangue backfill material. Furthermore, the creep results are further fitted using an empirical formula approach. Finally, the power law constitutive model for creep is obtained in double logarithmic coordinates. The filling and mining of gangue in mining areas hold significant engineering significance.

## Three-directional compression test for gangue backfills

### Test materials

**Figure 1:**
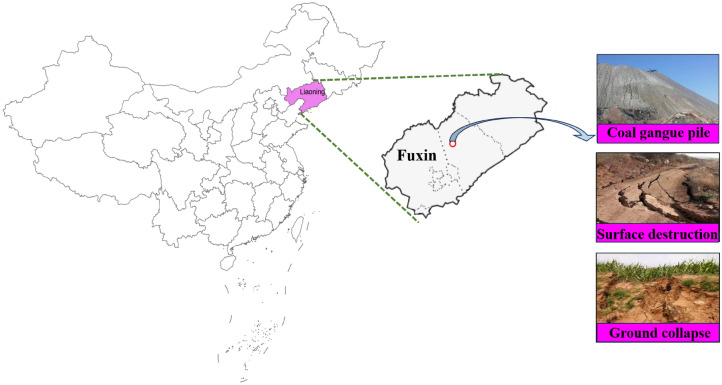
Geographical location of material source.

The gangue backfill material used in this experiment is from the gangue field of Fuxin City in Fig. 1, and the samples are prepared in the laboratory (crushing, screening, etc.) to obtain single particle size graded gangue, discontinuous graded gangue, and continuous graded gangue, respectively. The mechanical properties of the gangue backfill material were tested in the laboratory, and the basic parameters of the final gangue filling material were shown in Table 1.

**Table 1:** Basic parameters of gangue materials.

Property	Compressive strength (MPa)	Tensile strength (MPa)	Elastic modulus (MPa)	Particle size (Mm)
Sandstone	36.2	3.39	24.8	< 60

#### Single particle size graded gangue as shown in Fig. 2.

**Figure 2:**
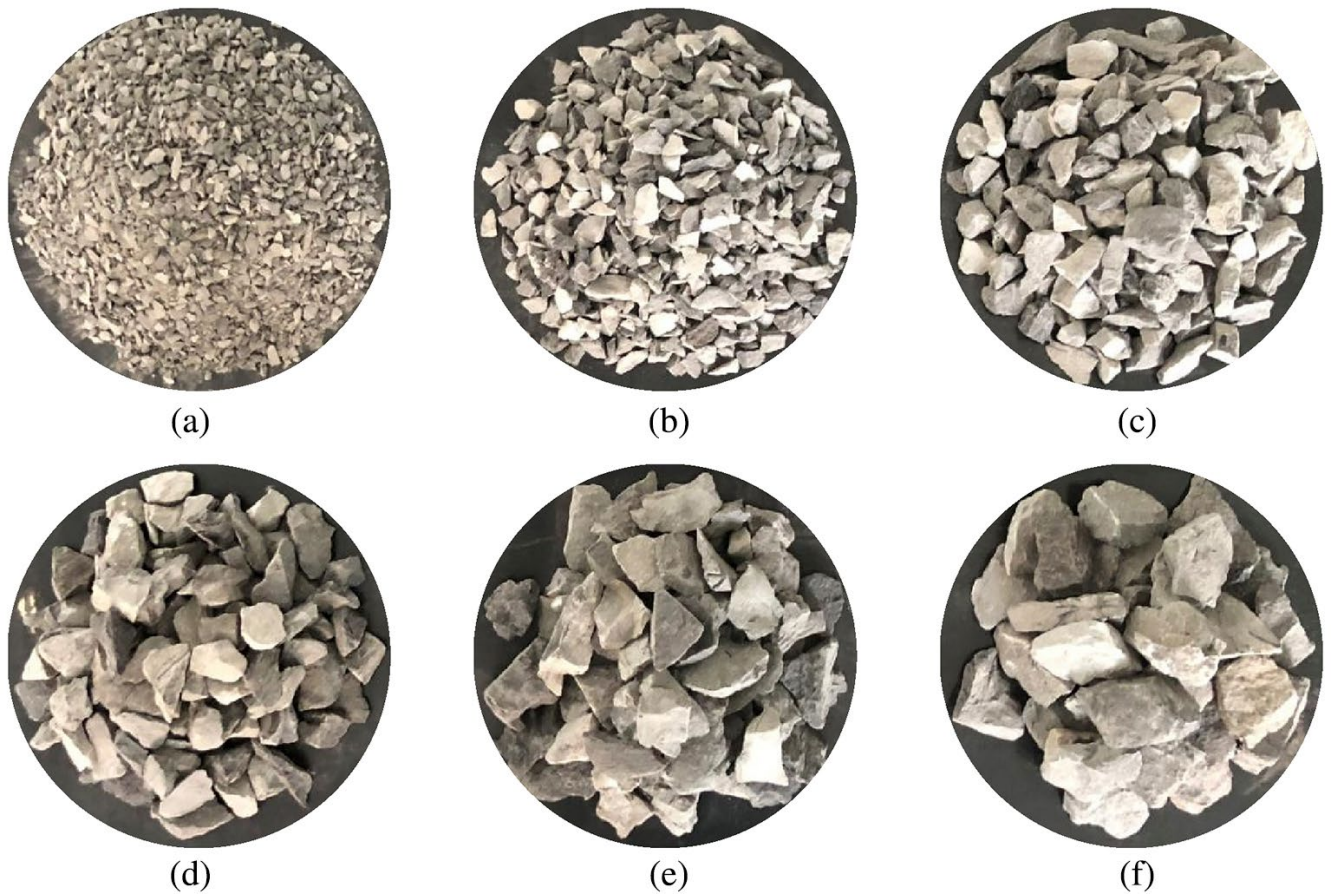
Different particle size graded gangue samples.

#### Continuously graded gangue

Using Talbot’s theory based on the continuous grading curve^33,34^, the gangue content was calculated within each particle size range in the grading, resulting in a continuous graded gangue in Table 2.

**Table 2:** Proportion table for continuous gradation gangue samples.

Talbot coefficient *n*	*P*_*x*_/%					
	0 ~ 10 mm	10 ~ 20 mm	20 ~ 30 mm	30 ~ 40 mm	40 ~ 50 mm	50 ~ 60 mm
*n* = 0.3	58.42	13.5	9.31	7.32	6.13	5.32
*n* = 0.4	48.84	15.6	11.35	9.24	7.94	7.03
*n* = 0.5	40.82	16.92	12.97	10.94	9.64	8.71
*n* = 0.6	34.13	17.6	14.25	12.43	11.23	10.36
*n* = 0.7	28.53	17.82	15.21	13.73	12.73	11.98

#### Discontinuously graded gangue

Gangue particles were screened using a threshold particle size of 10mm. Particles larger than this threshold were considered coarse, while those equal to or smaller than the threshold were considered fine. And the fine particle content was set at 10%, 20%, 30%, 40% and 50%, forming the proportions of discontinuously graded gangue backfill material, denoted as P.

### Test equipment

**Figure 3:**
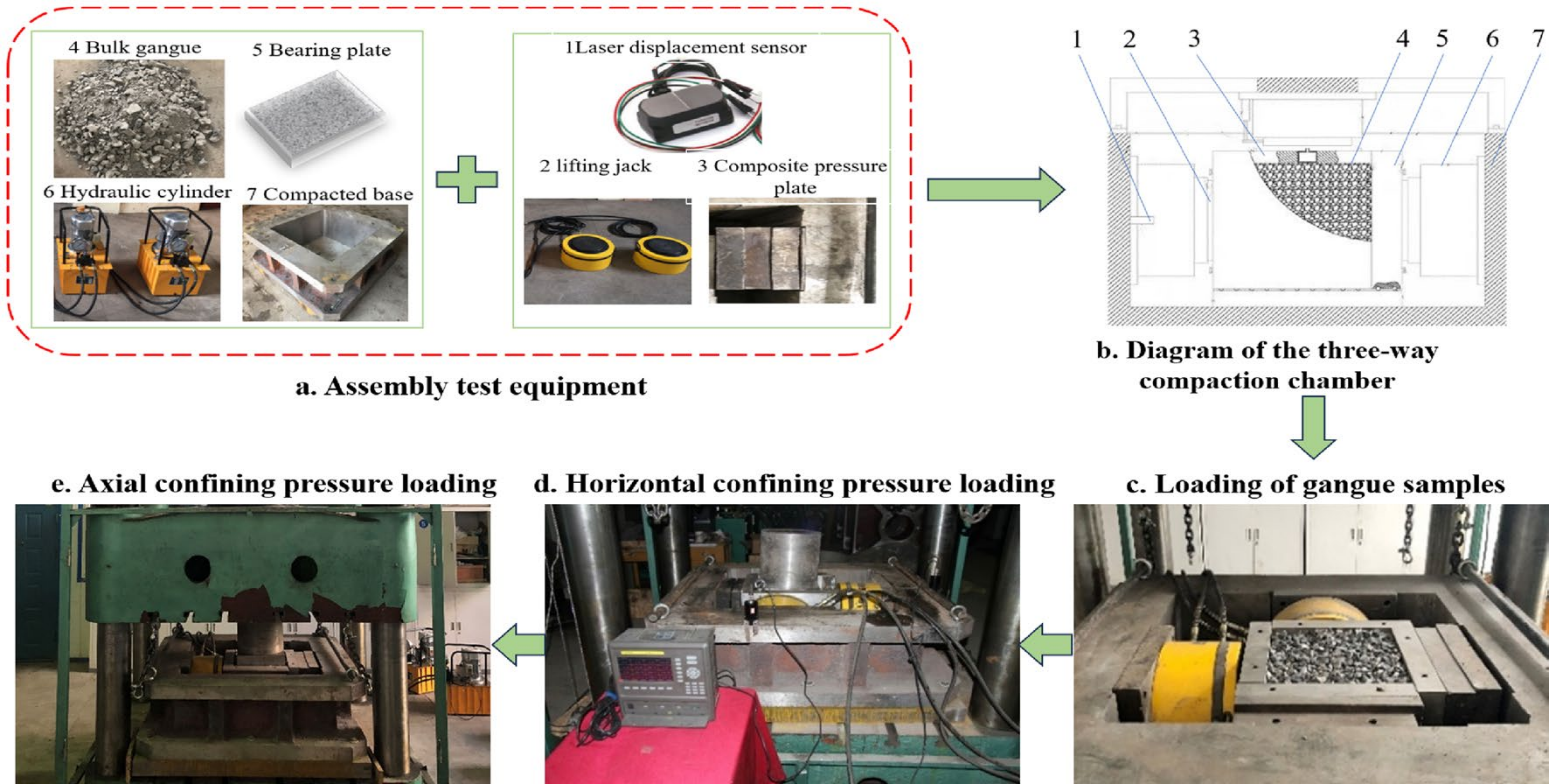
Three-directional compaction chamber composition and test flow chart.

**Figure 4:**
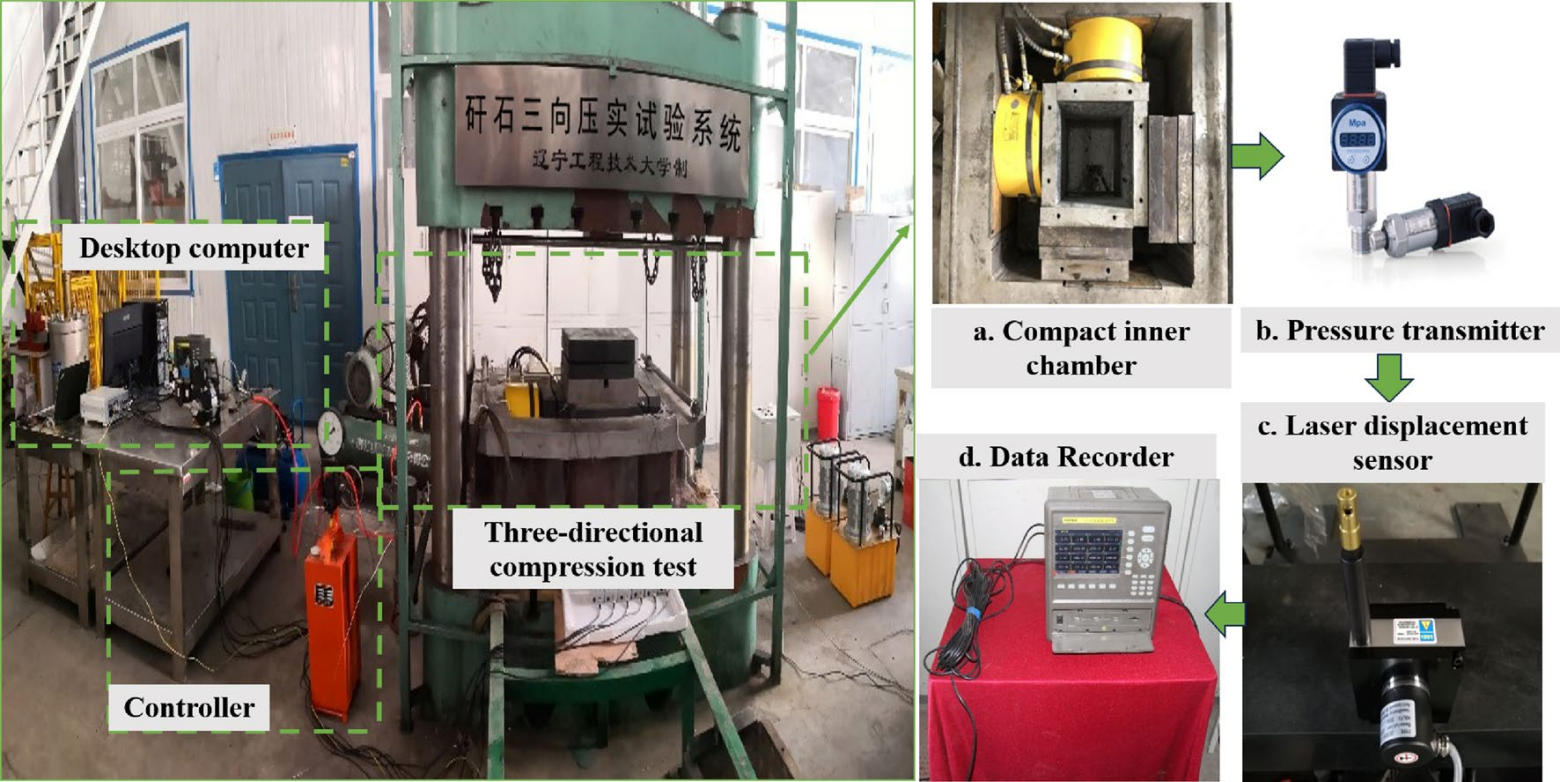
Three-directional compaction test system for gangue filling materials.

As existing true triaxial equipment is primarily designed for materials with a certain cohesion, such as rocks and soils, it is not suitable for studying the compaction of gangue^35^. As shown in Figs. 3 and 4, in this study, a large-scale, three-directional compression test system was developed, consisting of three main components: a stress application system for applying axial and horizontal stresses, a three-directional compaction chamber for filling gangue backfill materials, and a data monitoring and collection system for recording experimental data.

#### Stress loading system

The axial load system uses a computer-controlled electro-hydraulic servo compression testing machine with a maximum load of 5000 KN, a travel range of 1600 mm, a load control speed range of 0 to 80 KN/s, and a table size of 1.6 m × 1.6 m, which meets the required specifications.

#### Three-directional compression chamber

Three-directional compression chamber consists mainly of a horizontal loading plate, an axial loading plate, steel columns to transfer the axial load and the compaction chamber floor. The net dimensions of the compaction chamber are 400 mm × 400 mm × 400 mm. The thickness of the side load plate is 60 mm, the thickness of the axial load plate is 60 mm, the diameter of the section of the steel column carrying the axial load is 300 mm and the height is 100 mm. The lateral loading plate, the axial loading plate and the steel column transmitting the axial stress are all made of 45 # steel. The Young's modulus is 210 GPa and the Poisson's ratio is 0.35.

#### Data monitoring and acquisition system

The data acquisition system consists of pressure transmitter, guyed displacement sensor, laser displacement sensor, data recorder and desktop computer. The pull-wire displacement sensor is fixed to the servo press and the stroke can reach 500 mm. It can monitor the displacement of the press in real time, monitor the change in oil pressure in the oil pressure pump, and obtain the horizontal tension provided by the flat jack by conversion.

### Test scheme

The experimental program is shown in Table 3. The gangue creep loading method involves stepwise loading with a horizontal stress. In the experiment, each level of loading is stabilized for a period before proceeding to the next level of loading. The choice of test loads is determined by the level of stress. The loading time t of each stage is constant. The stress level L is divided into four levels^36^. After consolidation compaction of the gangue samples, creep tests were initiated. First, the confining pressure was applied to the predetermined value. Axial pressure was then applied according to the stress level. When the applied confining pressure reached the value specified in the test plan, the axial pressure was kept constant, and each loading level lasted for 6 h.

**Table 3:** Three-directional compression creep test scheme for gangue.

Serial number	Confining pressure *σ*_3_/MPa	Stress level *L*				Particle size range
1	2	0.2	0.4	0.6	0.8	Single particle size graded gangue 20–30 mm, 40–50 mm Discontinuously graded gangue fine20%. 50% Continuously graded gangue *n* = 0.3, 0.5
2	4	0.2	0.4	0.6	0.8	
3	6	0.2	0.4	0.6	0.8	
4	8	0.2	0.4	0.6	0.8	

### Test procedure

The test is divided into four steps: assembling the test apparatus, filling the gangue specimen, setting the confining pressure load, and applying the axial load.

1. Assemble each part of the test apparatus according to the design system and connect the stress and displacement monitoring devices. To avoid errors, apply an even layer of lubricating oil around the compaction chamber in advance to prepare for material loading.
2. After thoroughly mixing the three-directional graded specimens, place them in layers in the three-directional compaction chamber. Tests filled with gangue with a tool to scrape to the top of the chamber level, to ensure that each group of tests have the same amount of backfill material. Once filled, place the cover plate directly over the three-directional chamber.
3. Adjust the pressure machine so that it touches the upper cover plate, start the hydraulic pumping station, the displacement sensor and the data logger. Apply a horizontal load to the compaction chamber until the predetermined confinement pressure is reached, then adjust the jack to a stable condition.
4. Start the compression machine, control it to apply load until the lower surface of the axial load plate is in the same horizontal plane as the upper surface of the lateral load plate of the triaxial compaction chamber, and then zero the compression machine. Finally, control the computer to load according to the preset loading rate (1 KN/s), and stop loading when the requirements are met. This experiment was performed under rate-certain conditions.

## Analysis of three-directional compression results of gangue

### Analysis of different particle size test results

**Figure 5:**
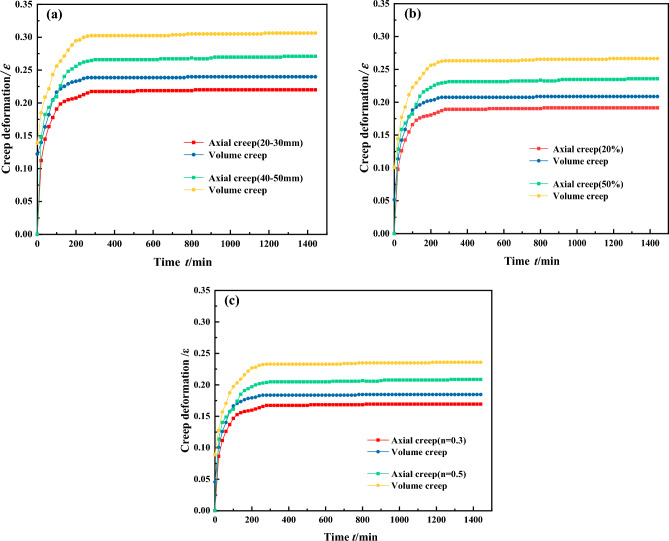
Compaction creep characteristic curve of gangue with different particle sizes (2 MPa).

As shown in Fig. 5, the results of the gangue creep grading load curve were processed to obtain the gangue creep deformation characteristic curves under different grain size conditions at a confining pressure of 2 MPa.

It can be seen from Fig. 5 that, samples with different particle sizes of gangue experienced two stages: stress compaction and stable creep. When the confining pressure is constant at 2 MPa. In 0–400 min, with the passage of time, the compaction deformation of different gangue types shows an initial increase followed by stabilization, and the creep deformation characteristics of gangue with different gradations show non-linear growth. During the compaction process, the voids between the gangue particles of different sizes are continuously compacted. During the creep stabilization stage after 400 min, gangue with a single particle size distribution exhibits the maximum creep deformation. As shown in Fig. 5a, the creep deformation of gangue with a single particle size of 20–30 mm increases from 0.237 to 0.24 at this stage, while the deformation of gangue with a single particle size of 40–50 mm increases from 0.302 to 0.306. The larger the particle size, the greater the creep deformation of the gangue sample. The reason for this phenomenon is that during the constant loading process, the gangue particles are further fragmented and there is a noticeable adjustment in the local pore structure, ultimately leading to the occurrence of this phenomenon.

As shown in Fig. 5b, for discontinuous graded gangue, the creep deformation is also affected due to the different content of fines grain. When the content is 20%, the creep change is generally small^37^. When the content reaches 50%, the creep deformation increases significantly. This is because as the fines increase, the voids between the gangue particles are gradually filled and the compressible voids between the particles become smaller and smaller. As shown in Fig. 5c, for continuously graded gangue samples, both axial and volumetric creep deformations show the least variation. This indicates that the particles are in close contact with each other and that there is minimal void space. The clamping effect is evident during the compression process. The fewer internal voids and cracks there are in the sample, the better its compaction stability under sustained load.

Consequently, we get that the magnitude of creep deformation growth rate is: single particle size gradation > discontinuous gradation > continuous gradation. At this stage, the axial stress of the specimen remains unchanged, affected by the lateral deformation of gangue material under lower peripheral pressure, the compaction deformation increases slowly with time, and the volume creep deformation of all types of gangues is greater than the axial creep deformation.

### Analysis of different stress test results

**Figure 6:**
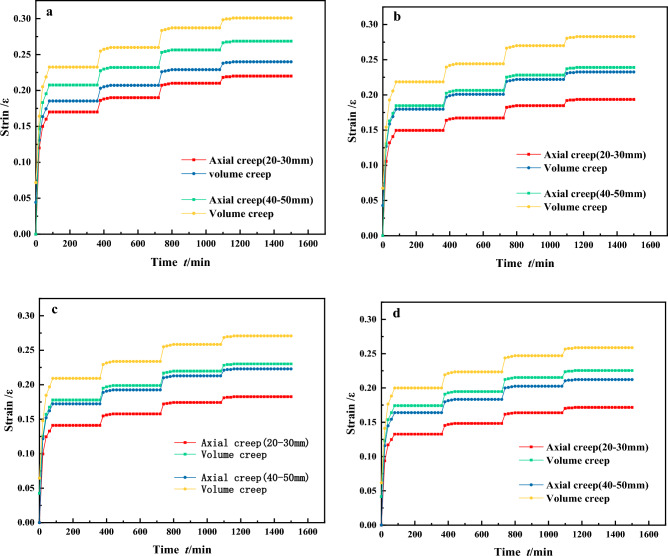
The strain–time relationship curve for a single particle size graded gangue.

As seen in Fig. 6, under different stresses, the stratification of single particle size graded samples is more pronounced and shows a step-like increase. This indicates that there has been a corresponding change in particle arrangement, accompanied by significant particle compression, crushing and partial particle disintegration. As the stress level increases, the gaps between the particles gradually decrease, leading to sliding and rolling of the particles to overcome frictional resistance. This movement causes the particles to reach a relatively stable position, leading the single particle size graded samples to eventually enter the creep stabilization phase. According to Fig. 6a,b, under the same stress conditions, gangue with a larger particle size exhibits a greater degree of compressive deformation. The final deformation of gangue samples with a single grain size of 20–30 mm is significantly less than that of samples with other grain sizes. In addition, the compressive deformation tends to stabilize more quickly for smaller grain size gangue samples. This is due to the relatively high voids between the large gangue particles, resulting in a greater compression space for the gangue sample during the compression process. The initial stage of axial stress loading with the same confining pressure. The axial creep and volumetric creep of the gangue sample both increase rapidly within the first 0–200 min, with the 40–50 mm single particle size grading showing significant deformation^38^. The axial deformation reaches 0.207, and the volume deformation reaches 0.232. The deformation of the gangue sample with a single particle size of 20–30 mm is rapid, with axial creep deformation reaching 0.172 and volumetric creep deformation reaching 0.185. The volumetric deformation is more pronounced than the axial deformation. Most of the deformation of the gangue sample is complete by 200 min and the creep deformation rate tends to 0. After continued loading, the gangue sample deforms under the action of external forces, with varying degrees of increase in both axial and volumetric creep deformations. Finally, at the L = 0.8 horizontal stage, the gangue sample reaches the creep stabilization stage with an axial deformation of 0.22, an increase of 0.01 compared to the previous stress stage, and a volumetric deformation of 0.239, an increase of 0.011. At this stage, after compaction through the first three stress levels, the voids between the gangue specimens have been significantly reduced, resulting in increased density. The compressive deformation of the gangue specimens is not as pronounced as it was during the initial stress stage. If greater compressive deformation of the gangue is desired, the stress must be increased, forcing the gangue to undergo continuous compression and fragmentation under higher pressure. The crushed gangue continues to fill the surrounding small voids, resulting in a further increase in gangue density and a gradual reduction in the magnitude of gangue compression deformation. The rates of axial and volumetric deformation decrease rapidly, gradually approaching equilibrium. Based on this analysis, it is suggested that creep deformation of continuously graded gangue samples occurs mainly at low stress levels, and this pattern is similarly observed for other grading conditions.

**Figure 7:**
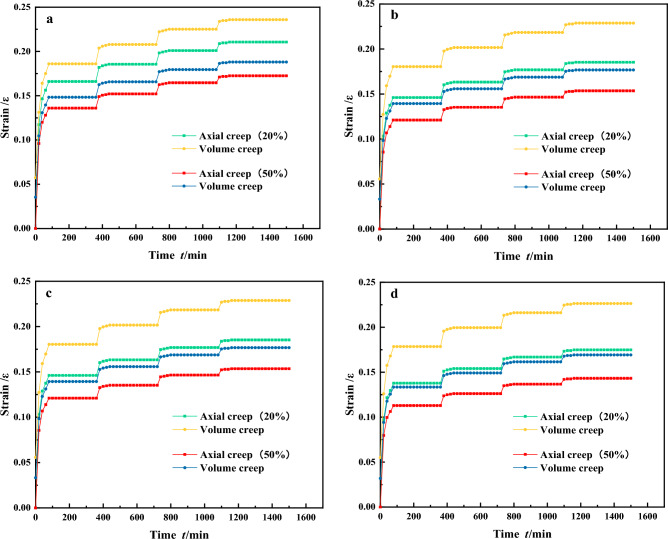
The strain–time relationship curve for discontinuously graded gangue.

For discontinuously graded gangue samples as shown in Fig. 7. Under different stresses, the stratification of discontinuously graded samples is pronounced and shows a stepwise increase. Under four different loading conditions, the axial deformation of the specimens is consistently smaller than the volumetric deformation. In addition, the gangue with a lower fines content shows a greater compressive deformation under the same stress load. The final deformation of the P = 50% discontinuous gangue sample is less than the deformations observed in samples with other different fines contents. In addition, the higher the fines content in the discontinuous graded gangue samples, the faster the compressive deformation tends to stabilize. This is due to the rapid stress concentration caused by the fines filling the gaps between the particles, leading to a faster achievement of creep stability. At the initial stage of axial stress loading under the same confining pressure, deformation occurs in the discontinuously graded gangue samples within 0–200 min. Among them, the discontinuous graded gangue with P = 50% deforms the fastest, with axial deformation reaching 0.139 and volumetric deformation reaching 0.148. For P = 20%, the deformation is the largest, with axial deformation reaching 0.166 and volumetric deformation reaching 0.185, and its volumetric deformation is more pronounced than the axial deformation. Around 200 min, most of the deformation in the gangue samples is complete and the creep deformation rate gradually approaches zero. The gangue samples continue to deform. Finally, at the L = 0.8 horizontal stage, the gangue specimens enter the creep stabilization phase. The axial deformation is 0.21, an increase of 0.012 compared to the previous stress stage, and the volumetric deformation is 0.235, an increase of 0.01. It can be observed that when the stress is applied to the fourth stage, the creep deformation rate of the gangue samples with different fines content is relatively slow and eventually stabilizes. When the fines content of the gangue sample is low and the grading is discontinuous, an increase in the fines content is beneficial for filling the voids between the coarse particles, resulting in a significant reduction in the compressible space of the gangue sample. When the fines content reaches 50%, the excess fines are distributed among the coarse particles of the gangue, reducing the frictional resistance to mutual displacement of the gangue particles. meanwhile, the creep deformation of the gangue sample with discontinuous gradation occurs mainly at low stress levels, as indicated by the early stages of stress loading.

**Figure 8:**
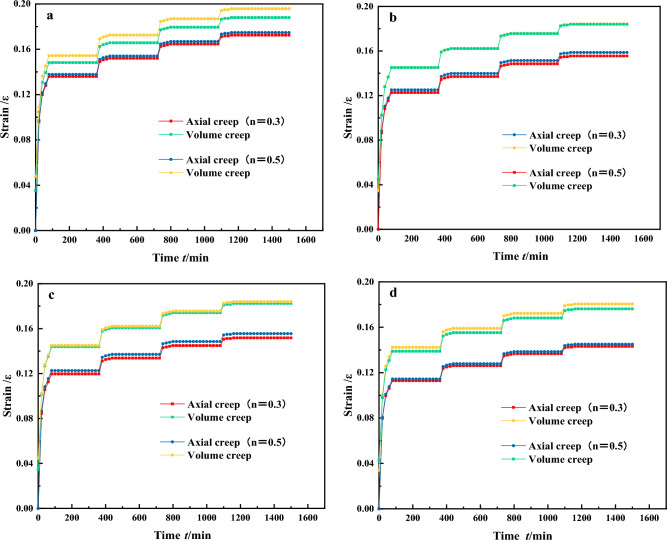
The strain–time relationship curve for continuously graded gangue.

For continuous gradation gangue samples as shown in Fig. 8. The compressive deformation curves of backfill materials with different continuous gangue gradations show slight differences. At the same level of loading stress, the creep deformation curves of the continuous gradation gangue samples are close to each other. The creep curves in Fig. 8b,c shows remarkable consistency. At the lowest loading stress level of 0.2, the creep curves show rate attenuation after compression deformation. After a certain period, the deformation rate gradually decreases to the creep stabilization stage with a rate of 0, and the creep deformation ceases to change. At the highest stress level of 0.6, the creep deformation curve experiences a rapid rate decay after undergoing instantaneous compressive deformation. After a period, it finally reaches the stable state of creep compression. The gaps between continuous graded gangue particles can be effectively filled by gangue particles of different diameters. Therefore, the deformation of continuously graded gangue samples is relatively stable, with a smaller deformation range than that of single particle size and discontinuously graded gangue samples. The creep deformation of gangue specimens with continuous grading can be seen to occur mainly at low stress levels.

### Analysis of different confining pressure test results

Horizontal comparison of the results in Figs. 6, 7 and 8, allows us to distinguish the axial creep and volumetric creep characteristics of gangue samples at different confining pressures. The analysis shows that the initial stages of triaxial compression processes for continuously graded gangue under different confining pressures are not significantly different. As shown in Fig. 8, the development trend of stable deformation regularity of continuous graded gangue specimens under different confining pressure conditions is the same. This is because the continuously graded gangue contains particles of different sizes, allowing different voids to be filled. The continuously graded gangue sample with n = 0.5 exhibits better initial void filling than the others gangue sample. After the load is applied, it first undergoes creep deformation and the duration of void compression is relatively short, quickly passing to the next stage. As the confinement pressure increases, gangue samples with the same gradation index reach the next stage more quickly. Analyzing the gangue samples with continuous grading index n = 3, it is observed that at a confining pressure of 2 MPa, the strain at the stable deformation stage is about 0.14, whereas at a confining pressure of 8 MPa, it is about 0.11. This is influenced by the effect of the confining pressure on the initial particle voids. The higher the confining pressure, the lower the initial void ratio, resulting in a better initial compaction density, and the same principle applies to other gangue gradations tested.

As shown in Fig. 6, when analyzing the single particle size gradations of the gangue samples under different confining pressures, it can be seen from the time-strain curves that the gangue samples with a single particle size of 20–30 mm reach the creep stability stage fastest under all four confining pressures. At a confining pressure of 2 MPa, the strain reaches about 0.17 at the initial stable stage and enters the creep stable stage at a strain of about 0.21. In contrast, the strains for the other groups range from 0.23 to 0.29. At a confining pressure of 8 MPa, the strain reaches approximately 0.13 at the initial stable stage and enters the creep stability stage at a strain of approximately 0.17. For the other groups, strains range from 0.21 to 0.25 and the evolution trends of the curves are similar before reaching the initial stable stage, with the curves essentially overlapping. This is due to the significant influence of the confining pressure on the initial compaction of the loose gangue material. The higher the confining pressure, the faster the single particle sized gangue samples reach the stable state. In addition, due to the uniformity of the single particle size graded gangue samples, it is difficult to compact the interparticle voids, resulting in significant deformation before initial stability is reached. The smaller the particle size, the smaller the change in compaction. At the same pressure, higher compaction is easier to achieve and tends to stabilize.

For the discontinuous graded gangue in Fig. 7, the evolution pattern of the time-strain curve is the same for the four confining pressure conditions^39^. When P is 20%, at a confining pressure of 2 MPa, the gangue sample reaches the initial stable state at a strain of about 0.13. It enters the creep stable state at a strain of about 0.2. At a confining pressure of 8 MPa, the gangue sample reaches the initial stable state at a strain of about 0.13 and enters the creep stable state at a strain of about 0.17. When P is 50%, under a confining pressure of 2 MPa, the gangue sample reaches the initial stable stage at about 0.13, while other groups range from 0.14 to 0.18. At a strain of about 0.17 it enters the creep stable stage and other groups range from 0.18 to 0.23. At a confining pressure of 8 MPa, it enters the initial stable state at about 0.11, while other groups are at 0.13 and 0.17. At a strain of about 0.14 it enters the creep stable stage and other groups range from 0.16 to 0.22. This indicates that the higher the confining pressure, the faster the discontinuous gangue will reach the stable deformation stage. Increasing the confining pressure increases the resistance of the discontinuous gradation gangue to deformation, making it more stable over time.

From the comparative analysis of Figs. 6a,d, 7a,d and 8a,d, it can be observed that during the initial stable phase of the gangue, the axial creep deformation of the gangue samples is generally smaller than the volumetric creep deformation. This indicates that there is a significant influence of confining pressure in the early stages of creep deformation. Furthermore, under low stress conditions, the deformation induced by creep is mainly volumetric compression. This indicates that volumetric compression is the primary manifestation of deformation in the gangue creep process. Lateral contraction of gangue specimens under confining pressure is an important factor in the generation of creep deformation. Upon completion of the axial loading, both the axial and volumetric strains of the gangue specimen gradually come to a standstill. However, at this point the rate of change of the axial strain is significantly greater than that of the volumetric strain. In addition, cracking noises were heard during the creep loading process. Analysis suggests that in the early stages of creep loading, only a small number of gangue particles fracture because the axial load does not reach the load required for gangue particle fracture. As the stress level increases, the gangue continues to undergo creep deformation, progressively breaking into smaller particles. The particles rearrange and reorganize to occupy voids and experience mutual compression, ultimately leading to this phenomenon.

## Characteristics of load-bearing crushing in gangue fill material

### Analysis of gangue fragmentation rate under different gradation conditions

**Figure 9:**
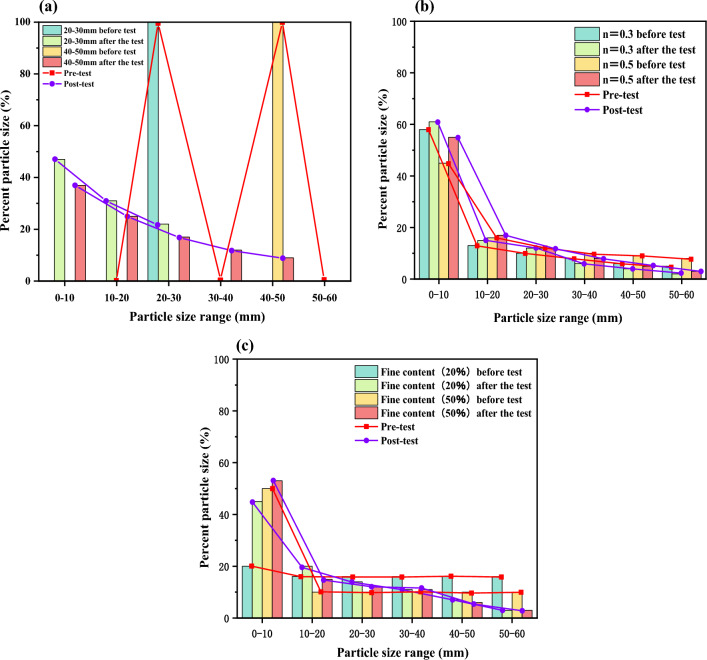
Particle size distribution under different grading conditions before and after creep compaction (4 MPa).

It can be observed in Fig. 9 that the changes in particle content before and after the triaxial compaction creep test of gangue samples are significantly influenced by the gradation. Analyzing Fig. 9a, it is evident that when the stress on the gangue sample is compressed to 4 MPa, there is a noticeable and intense fragmentation in the single size gangue samples. A comparison shows that the degree of fragmentation is higher for larger single size gradations. Its primary manifestation is the continuous decrease in the mass fraction of large particles and the appearance and increase in the mass fraction of small particles. Looking at the particle size distribution curves before and after crushing, the small particles after crushing show an approximately linear distribution. As the applied stress increases to the critical fracture stress intensity, larger particles are continuously fragmented to form smaller particles that complement the particle distribution in the initial Discontinuous grading interval. For the 20–30 mm gangue sample graded by size, the most significant increase is in the 0–10 mm grading, where the 0–10 mm particle size reaches the highest proportion at 47%. In the 10–20 mm grading with interruption, the 10–20 mm particle size accounts for 31%, while in the initial 20–30 mm graded gangue sample it decreases to 78%. After crushing, the particle size distribution is only 22%. For the 40–50 mm single gradation sample, it is looser after crushing compared to the 20–30 mm gradation sample. In the 0–10 mm grading with interruption, the 0–10 mm grain size increases significantly and reaches a proportion of 37%. While the initial gradation of the 40–50 mm gangue sample decreases drastically by 91%, the proportion after crushing is only 9%. The initial gradation interval lacks small particles, resulting in poor stability and less resistance to deformation failure. Therefore, the single particle gradation is more prone to re-crack. As the voids within the sample are compressed and the internal particle structure is adjusted and optimized, the skeletal framework of larger particles undergoes continuous strengthening under the influence of stress. This leads to a concentration of stress on the particles within the supporting framework, resulting in a significant amount of fragmentation. The gangue samples have fragmented into particles of different sizes. When a new equilibrium is reached between the large particles and the particles, it becomes difficult for the larger particles to undergo further fragmentation.

Figure 9b shows that the continuous gradation gangue sample exhibits more pronounced fragmentation when compressed to a stress of 4 MPa. The degree of fragmentation in the two continuous gradation gangue samples is relatively close. Based on the particle size distribution curve before and after fragmentation, the particle size distribution after fragmentation shows an approximately linear pattern. After the fragmentation experiment, there were changes in the particle gradation compared to the initial gradation. The proportion of larger particles decreased while the proportion of smaller particles increased, with a greater increase observed in the finer particle size groups. This is because the larger particles are crushed by compression, forming smaller particles that subsequently complement the initial continuous gradation of smaller particle groups. In the case of continuous gradation (n = 0.3), there is an increase in the proportion of particles in the 0–30 mm size range, with an increase of between 2 and 3%. Conversely, the proportion of particles in the 30–60 mm range decreases, particularly in the 50–60 mm range, where the decrease is as much as 2%. In the case of continuous gradation (n = 0.5), there is a slight increase in the proportion of particles in the 0–30 mm size range. The proportion of particles in the 20–30 mm size range remains almost unchanged before and after fragmentation. Conversely, the proportion of particles in the 30–60 mm size range decreases, with the 50–60 mm size range reduced to only 3% of its original value. Overall, the proportions of particle sizes in the continuous grading gangue samples change insignificantly before and after fragmentation. The analysis suggests that continuous gradation compensates for the shortcomings of single size gradation. Gangue with different particle sizes can complement each other structurally during the compaction process, with small particles filling the voids between larger particles. This results in improved compaction and enhanced compaction performance of continuous gradation gangue samples.

As shown in Fig. 9c, when the gangue specimen is compressed to a stress of 4 MPa, the discontinuous gradation gangue specimens exhibit significant fragmentation. The degree of fragmentation is relatively similar between the two types of discontinuous gradation gangue specimens. When comparing the two discontinuous gradation conditions, the reduction is most significant for the discontinuous grading with P = 20%. In the 0–10 mm particle size range, there is a significant increase in the occurrence of small particles, resulting in a significant increase in the particle size percentage from 20 to 45%. There is a decrease in the percentage of particle sizes in the 10–50 mm range, with reductions ranging from 2 to 5%. The particle size range of 50–60 mm shows the most significant reduction, from 16% to only 3%. For the discontinuous gradation (P = 50%), the reduction is the smallest. Analysis suggests that the crushing of the discontinuous gradation gangue samples increases the interaction forces and compression between the gangue particles. Larger, harder gangue particles are continuously crushed and fractured, further filling the voids and causing significant sample contraction. This is also related to the fines content and the gangue samples with a fines content of P = 50% show a relatively mitigate degree of fragmentation. Due to the surrounding effect of the fines filling, the compressive fragmentation capacity of the discontinuous gradation gangue samples is further enhanced. In comparison, this is also the reason for the significant change in particle size distribution before and after fragmentation in the case of fines content P = 20%.

**Figure 10:**
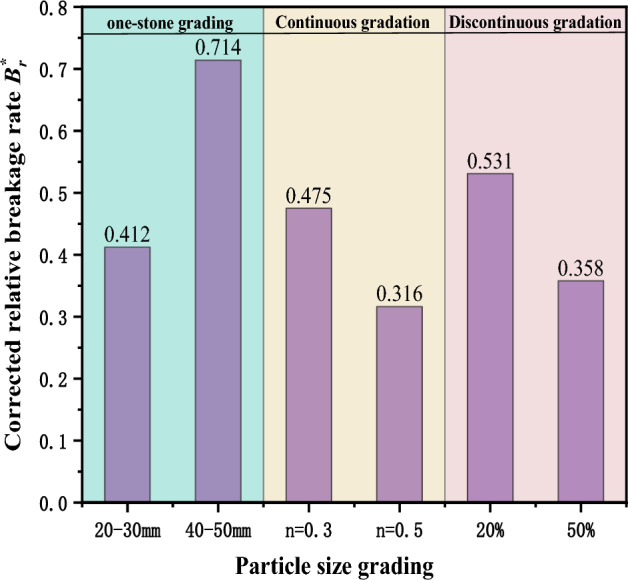
Fragmentation rate of gangue samples with different gradations. (Confining pressure 4 Mpa).

According to the adjusted relative fragmentation rate^40^, define the distribution of the adjusted relative fragmentation rates for different gradation samples is shown in Fig. 10.

The fragmentation rates of the gangue materials vary significantly between the different particle size gradations. For the single size gradation sample with a particle size of 40–50 mm, the adjusted relative fragmentation rate reaches a maximum of 0.714, while for the continuous gradation sample (n = 0.5) the lowest adjusted relative fragmentation rate is 0.316. For gangue samples with other particle size gradations, the adjusted relative fragmentation rates show little variation, ranging from 0.173 to 0.054. The analysis suggests that for the same stress (4 MPa), small particle size gangue samples experience a slow increase in stress during the initial loading phase, whereas large particle size gangue samples experience a rapid increase in stress during the initial loading phase, leading to the occurrence of crushing failure. As the proportion of large particle gangue in the sample increases, it becomes more susceptible to compression crushing and the degree of fragmentation is higher. For continuous gradation gangue samples, they inherently possess a stable load-bearing framework, making it more likely to meet the stability requirements of crushing compaction when subjected to loads. Hence, the stability of the structural load-bearing capacity is stronger, making it less prone to undergo compressive deformation. In addition, it effectively suppresses the generation of further gangue particle fractures due to the additional filling of voids between large particles by the crushed gangue under compression. Consequently, its adjusted relative fragmentation rate is the lowest.

### Analysis of gangue fragmentation rate under different confining pressures

**Figure 11:**
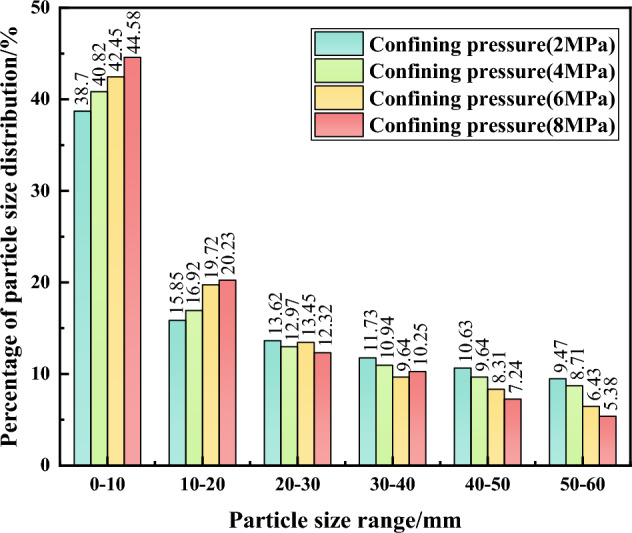
Particle size distribution of gangue samples under different confining pressures (continuous grading n = 0.2).

It can be seen in Fig. 11 that there is no significant variation in the particle size of the gangue samples during the triaxial compaction creep test. In the creep loading results, there is an overall decrease in the content of large gangue particles in the 40–60 mm particle size range, while there is an increase in the content of small gangue particles in the 0–20 mm particle size range. In the creep loading results, there is an overall decrease in the content of large gangue particles in the 40–60 mm particle size range, while there is a slight increase in the content of small gangue particles in the 0–20 mm particle size range, although both increases and decreases are relatively small. In the 20–40 mm particle size range, there is a slight overall decrease in gangue particle content. However, there is no apparent regularity with the confining pressure varies. Among these, the particle size range with the greatest reduction in content is 50–60 mm, with a reduction of 4.03%. The particle size range with the highest increase for small particles is 0–10 mm with an increase of 5.88%. This is followed by the 10–20 mm range with the second highest increase of 4.38%, while the 20–40 mm range shows minor variations which will not be discussed further. The particle size range with the highest increase for small particles is 0–10 mm with an increase of 5.88%. This is followed by the 10–20 mm range with the second highest increase of 4.38%, while the 20–40 mm range shows minor variations which will No further elaboration. The analysis suggests that as the confinement pressure increases, the large particle samples undergo continuous fragmentation, eventually reaching a new equilibrium state. The voids between the gangue particles gradually fill, reducing inter-particle friction and relative movement ceases. In addition, the smaller gangue particles are less susceptible to fragmentation, resulting in less variation and eventually a stable state.

**Figure 12:**
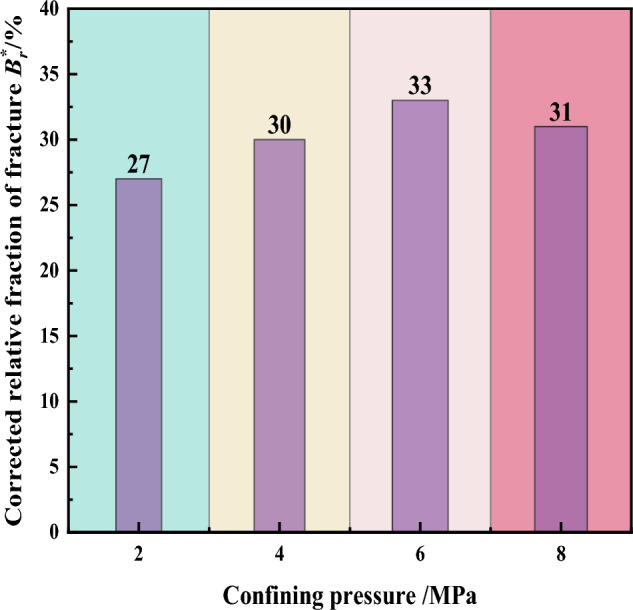
Crushing rate of gangue material under different confining pressure.

According to the adjusted relative fragmentation rate, define the distribution of the adjusted relative fragmentation rates for different gradation samples is shown in Fig. 12.

Different confining pressure conditions have some effect on the particle fragmentation of the gangue samples. As the confining pressure increases, the relative fragmentation rate of the gangue samples tends to increase and then decrease. When the confining pressure is 2 MPa, the adjusted relative fragmentation rate of the gangue sample is 27%. As the confining pressure increases to 4 MPa and 6 MPa, the relative fragmentation rates are 30% and 33% respectively. At a confining pressure of 8 MPa, the relative fragmentation rate of the sample instead decreases to 31%. The analysis suggests that at a confining pressure of 2–6 MPa, the gangue samples experience strong lateral confinement, resulting in continuous fragmentation of irregular edges and corners of gangue particles under sustained loading. As the confining pressure exceeds 6 MPa, there is a decrease in particle fragmentation within the gangue samples. This is attributed to the compact contact among gangue particles under the influence of confining pressure, limiting the space for particle fragmentation. This macroscopically manifests as a weakening effect leading to a reduction in fragmentation rates.

### Crushing creep mechanism of gangue fill material

**Figure 13:**
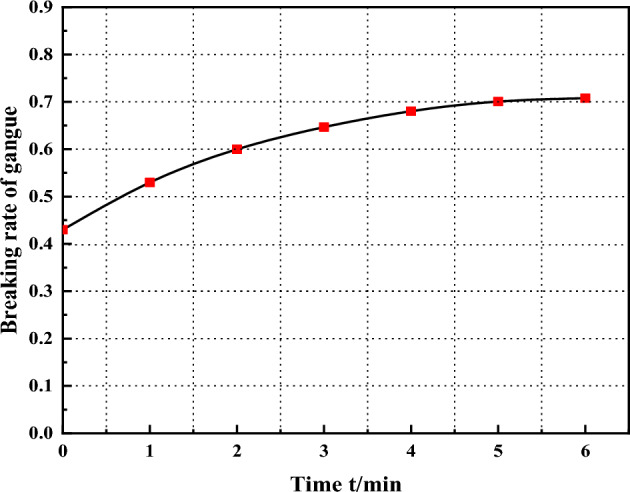
The relationship between the fragmentation rate of gangue particles and time.

The relationship between the fragmentation rate of gangue particles and time in Fig. 13. Gangue specimen under the action of external load, rapid deformation, which is due to the gangue specimen loose has not formed a complete and stable skeleton structure. As the lateral axial force continues to increase, the gangue compression produced by small particles of the gangue mutual extrusion movement leads to sliding dislocation, making the gangue crushing phenomenon is more and more obvious. However, the gap between the particles becomes smaller, making the adjacent particles more compact, resulting in a reduction in the volume of the specimen, when the movement between the particles to rebalance the position, the gangue particles rotate each other, moving and rearranging and other behaviors will be difficult to occur, the deformation of the samples is gradually slowed down, and ultimately, the specimen is in a new equilibrium state. The main factors that produce deformation in gangue-filled specimens are: the movement of particles under stress affects the macroscopic deformation of gangue-filled materials, and the other is the time-dependent creep mechanism that occurs in particles under sustained high stress. From this, it is known that particle crushing to fill the gap between the particles is the main reason for the creep deformation of gangue specimens.

**Figure 14:**
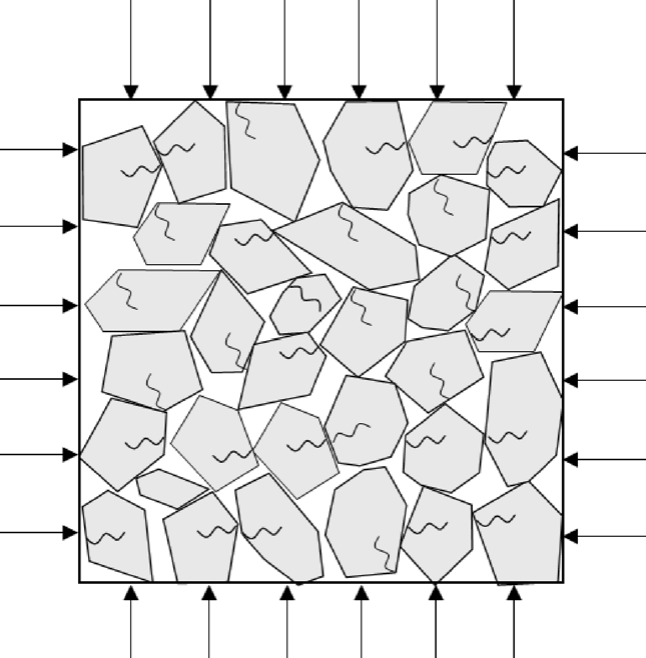
Compressibility and creep crush model of gangue.

From a Detailed View, as the triaxial loading of the gangue sample progresses, the contact and bite of the gangue particles leads to compression adjustments, resulting in a reduction in the void space within the sample. If the gangue fill is subjected to prolonged and complex environmental conditions in deep burial conditions, the internal fine cracks within the gangue particles will continue to propagate, resulting in stress concentration phenomena as shown in Fig. 14. During this period, the gangue sample experiences an increase in both microscopic particle fragmentation and macroscopic deformation until the adjustment of gangue particle positions and fragmentation reaches a new equilibrium. Therefore, the internal repetition of the above process in the gangue sample ultimately constitutes the process of fragmentation creep in the gangue.

## Creep power law constitutive model of gangue fill material

### Expression of the creeping power-law model

Due to the satisfactory fitting performance of the power-law model in both the early and late stages of deformation, and its relatively rapid deformation in the early stage, the power-law model is preferred, especially for the deformation under deep stress conditions in the granular fill material. Therefore, the power law expression is adopted as the creep model for the gangue fill material. In the experimental study of the creep characteristics of granular materials, a power law expression is used to elucidate the triaxial compressive creep behavior of the gangue backfill material as Eq. (1):

1 $$\varepsilon =a{\left(\frac{t}{{t}_{0}}\right)}^{b}$$

where t_0_ is the starting point in time during the creep test duration; a is the initial axial (or volumetric) creep of the gangue sample at t_0_; b is the axial (or volumetric) creep index of the gangue sample after t_0_.

### Creep curve of the gangue backfill material with double logarithmic coordinates

The value of t_0_ in Eq. (1), directly influences the initial creep a and the creep index. According to Reference^41,42^, the creep deformation of granular materials exhibits a linear correlation with time in a double logarithmic coordinate system. Investigate whether the axial strain (volumetric strain) and time curves of the gangue fill material under double logarithmic coordinates meet the requirements of the creep model and determine the relevant time parameters.

#### Creep time characteristics of gangue in double-logarithmic coordinates

**Figure 15:**
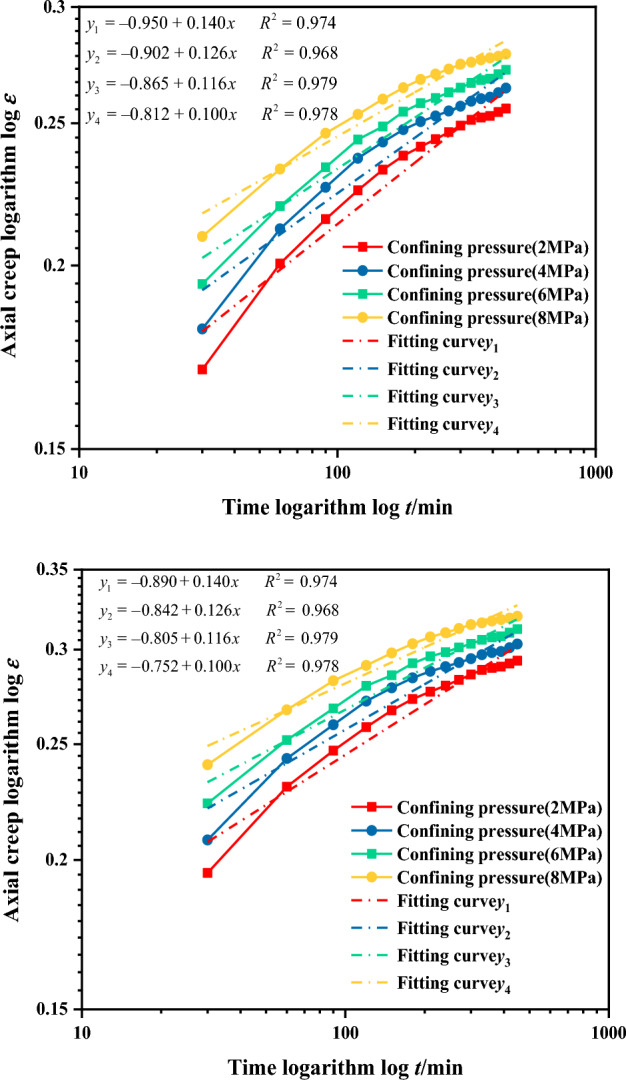
Creep time double-logarithmic coordinate curve of continuously graded gangue (n = 0.2).

From Fig. 15, it can be observed that the axial and volumetric creep of the gangue specimens in double-logarithmic coordinates have a non-linear relationship in the early stage and a linear relationship in the later stage with respect to time. Linear fitting of the curves in the figure shows that the slopes of the curves vary under different confinement pressures, ranging from 0.1 to 0.13. The fitting results are not satisfactory, with R2 values between 93 and 95%. The creep of the gangue samples in double logarithmic coordinates is approximately linear with time, making it suitable for fitting with a power law model.

#### The determination of the creep t_0_ moment

**Figure 16:**
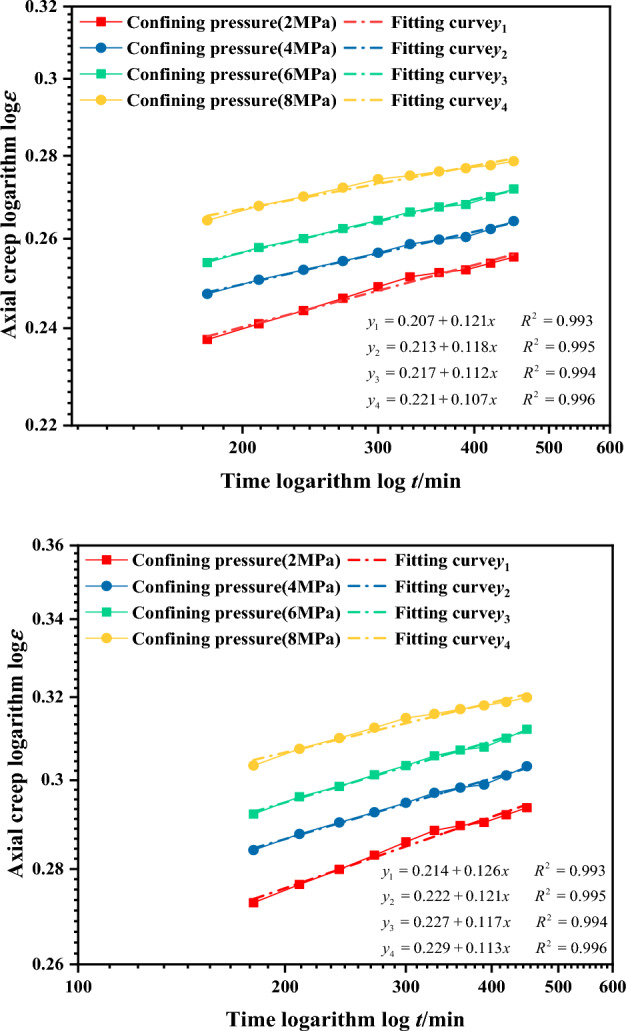
Creep-time double logarithmic coordinate curve for continuously graded gangue (*n* = 0.2, *t*_0_ = 180 min).

In Eq. (1), when t_0_ = 0 min, the axial (volumetric) creep curve changes steeply at a low stress level. Consequently, the initial creep increment obtained from the fitted creep-time relationship curve is uneven, resulting in an overall concave shape in the logarithmic coordinate curve. The creep deformation of the gangue backfill material mainly occurs within several years after filling, the adoption of the latter part of the experimental curve better characterizes the long-term creep deformation of the gangue backfill material, which is more in line with the actual behavior. After several experiments, when t_0_ = 180 min is selected, the linear fit of the latter part of the creep curve in the double logarithmic coordinate system shows good performance under different conditions. With R^2^ values ranging from 99.2% to 99.6%, it is determined that t_0_ = 180 min is the starting point for the duration of the creep test. The fitting results of the creep-time double logarithmic coordinate curve for the gangue under t_0_ = 180 min are shown in Fig. 16.

#### Creep parameter fitting

The creep time curve starting at t = 180 min is selected as the initial data for the relationship model solution. After reselecting the experimental points, a straight line is used for analysis. The initial creep value at time t = 180 min is taken as the axial (volumetric) initial creep (a) for the gangue material. The slope of the fitted line is taken as the gangue material axial (volumetric) creep index (b). The values of the creep related parameters for the gangue fill are shown in Table 4.

**Table 4:** The results of the creep related parameters for the gangue fill material are presented.

Confining pressure (MPa)	Axial initial creep a_1_	Axial creep index b_1_	Volume initial creep a_2_	Volume creep index b_2_
2	0.207	0.121	0.214	0.126
4	0.213	0.118	0.222	0.121
6	0.217	0.112	0.227	0.117
8	0.221	0.107	0.229	0.113

### Parameters of the power function axial creep model for gangue backfill material

Based on the power function expression and three-directional compression test results of the gangue fill, this study investigates the functional relationship between initial axial creep and creep index with stress level and confining pressure, with the aim of establishing an axial creep model.

#### Axial initial creep deformation a_1_

**Figure 17:**
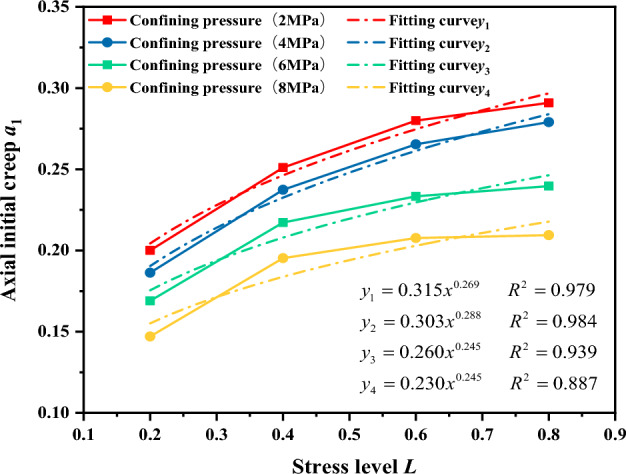
The relationship between axial initial creep deformation a_1_ and stress level L.

**Figure 18:**
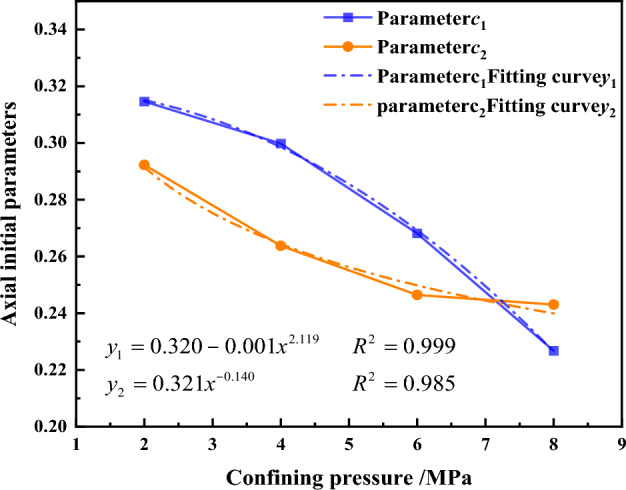
The relationship between other parameters of axial initial creep deformation (c_1_, c_2_) and confining pressure *σ*_3_.

In three-directional compression test, the stress level has a significant effect on the axial creep deformation of the gangue material. Therefore, when determining the expression for initial axial creep, the stress level is considered first, followed by the influence of the confining pressure. The relationships between initial axial creep a_1_ and stress level L and confining pressure *σ*_3_ were fitted using the corresponding parameters from Fig. 16 and Table 4. The fitting results are shown in Figs.17 and 18.

Figure 17 shows the relationship between the initial axial creep deformation a_1_ of the gangue material and the stress level L. As shown in the Fig. 17, for the same confining pressure, the initial axial creep deformation of the gangue material increases with a power-law exponent as the stress level increases. Therefore, the relationship between the initial axial creep deformation of the gangue material and the stress level is given as Eq. (2):

2 $${a}_{1}={c}_{1}{L}^{c2}$$

**Figure 19:**
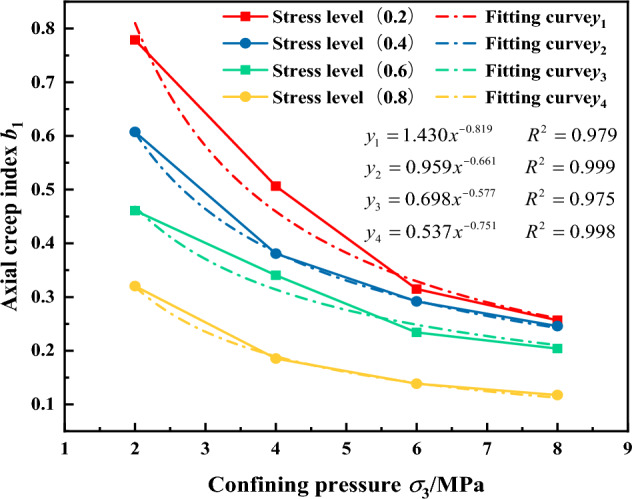
The relationship between the axial creep exponent b_1_ and the confining pressure *σ*_3_.

Based on the results of the initial axial creep deformation a_1_ and stress level L in Fig. 18. The relationship between other parameters of the initial axial creep deformation c_1_, c_2_ and the confining pressure*σ*_3_ has been established as shown in Fig. 19. The parameters c_1_, c_2_ and the confining pressure *σ*_3_ have a power function relationship. They are therefore expressed in terms of power functions, giving the relationship between the parameters c_1_, c_2_ and the confining pressure *σ*_3_:

3 $$\begin{array}{c}{c}_{1}=0.32-0.00114{{\sigma }_{3}}^{2.119}\\ {c}_{2}=0.32{{\sigma }_{3}}^{-0.14}\end{array}$$

Substituting the parameters c_1_ and c_2_ into Eq. (2), the expression for the initial axial creep deformation a_1_ of the gangue material is given as:

4 $${a}_{1}=\left(0.32-0.00114{{\sigma }_{3}}^{2.119}\right){L}^{{{0.32\sigma }_{3}}^{-0.14}}$$

#### Axial creep exponent b_1_

**Figure 20:**
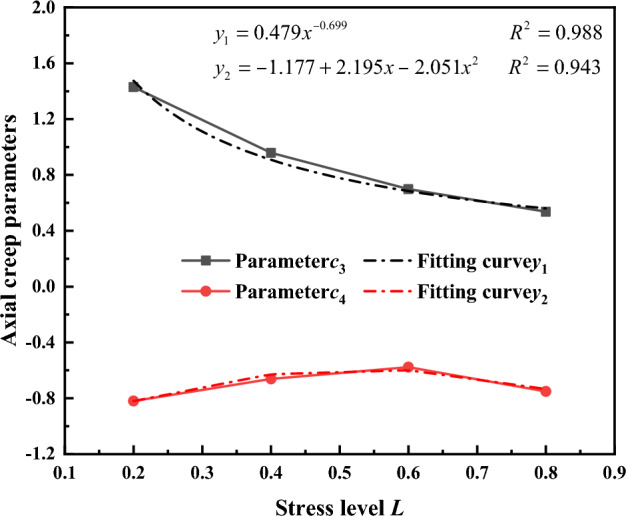
The relationship between the axial creep exponent b_1_ and the stress level c.

Based on the results of the gangue three-directional compression test, it is observed that the confining pressure is the primary factor influencing the axial creep exponent, while the stress level is a secondary factor. Therefore, the influence of the confining pressure is considered first, followed by consideration of the stress level. Fitting the relationships between the axial creep exponent b_1_ and the stress level L and the confining pressure *σ*_3_, gave the results shown in Figs. 19 and 20, respectively.

As show in Fig. 19, the relationship between the axial creep exponent b and the confining pressure c of the gangue material is evident. During the three-directional compression test, the axial creep exponent of the gangue material decreases noticeably with an increase in confining pressure. Similarly, the relationship between the axial creep exponent and the confining pressure can be described by the power function represented by the parameter "*b*_1_ = *c*_3_ *σ*_3_^*c*4^".

Based on the fit results of the axial creep exponent b_1_ and confining pressure *σ*_3_ in Fig. 20, the relationship between the other parameters *c*_3_, *c*_4_ and the stress level "L" of the gangue material axial creep exponent is established as shown in Fig. 19. The parameter c_3_ has a power function relationship with the stress level L, while c_4_ has a quadratic function relationship with the stress level L. Therefore, expressions are formulated using both a power function and a quadratic function, respectively. This gives the relationship between parameters *c*_3_, *c*_4_ and stress level L as:

5 $$\begin{array}{c}{c}_{3}=0.479{L}^{-0.698}\\ {c}_{4}=-1.774+2.195L-2.051{L}^{2}\end{array}$$

Substituting the parameters *c*_3_ and c_4_ into the power function* b*_1_ = *c*_3_*σ*_3_^*c*4^, the axial creep exponent of the gangue material is given by**:**

6 $${b}_{1}=0.479{L}^{-0.698}{{\sigma }_{3}}^{-1.774+2.195L-2.051{L}^{2}}$$

Substituting Eqs. (4) and (6) into formula (1), the expression for the axial creep power-law constitutive model is given by:

7 $${\varepsilon }_{rh}=\left(0.32-0.00114{{\sigma }_{3}}^{2.119}\right){L}^{{{0.32\sigma }_{3}}^{-0.14}}{\left(\frac{t}{180}\right)}^{0.479{L}^{-0.698}{{\sigma }_{3}}^{-1.774+2.195L-2.051{L}^{2}}}$$

### Parameters of the power function volume creep model for gangue backfill material

Using the volume creep value of the gangue sample at t_0_ = 180 min as the initial volume creep a_2_. Combined with the results of the three-directional compression creep test of the gangue backfill material. The initial volumetric creep and creep exponent as a function of stress level and confining pressure were analyzed, leading to a volumetric creep model.

#### Initial volume creep *ε*_*a*_

**Figure 21:**
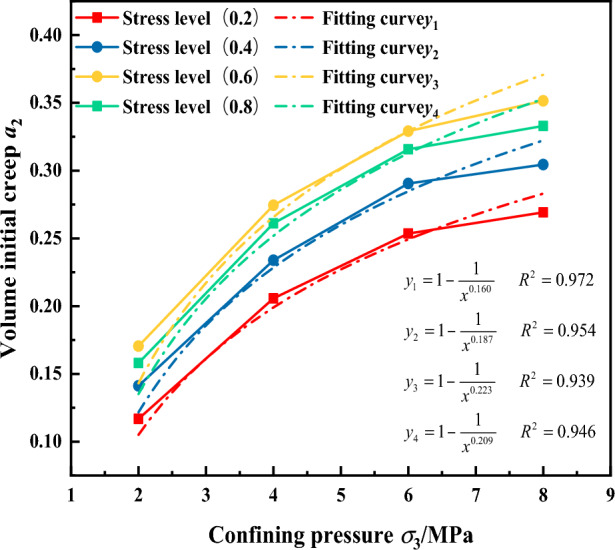
Relationship between initial volumetric creep a_2_ and confining pressure.

**Figure 22:**
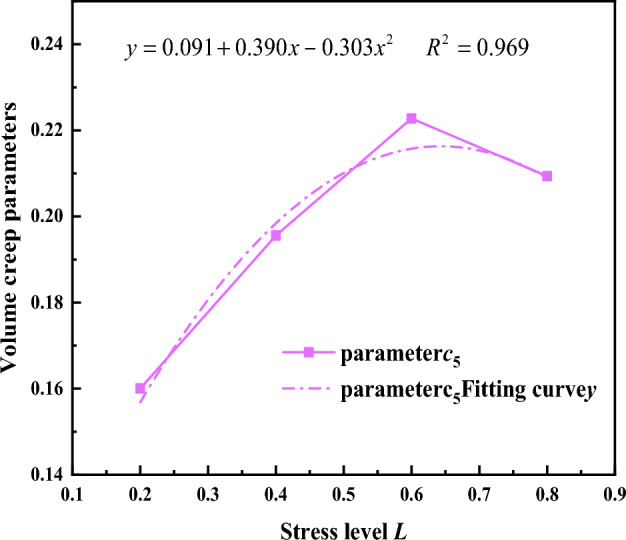
Relationship between volumetric creep parameters and stress level.

In the three-directional compression test, the confining pressure has a significant effect on the volumetric creep of the gangue material. Therefore, in determining the expression for the initial volumetric creep of the gangue material, the influence of the confining pressure is considered as a primary factor. Based on the results of the logarithmic relationship in Fig. 16, and the volumetric creep related parameters in Table 4, the relationships between the initial volumetric creep a_2_ and the confining pressure *σ*_3_, as well as the stress level L were fitted separately. The fitting results are shown in Figs. 21 and 22.

As can be seen in Fig. 21, illustrates the relationship between the initial volumetric creep a_2_ and confining pressure *σ*_3。_ The fitted equation from the graph shows that the initial volumetric creep exponent grows exponentially as the confining pressure increases. Therefore, the relationship between the initial volumetric creep of the gangue material and the confining pressure can be described by a power function as:

8 $${a}_{2}=1-{{{\sigma }_{3}}^{c}}^{5}$$

Based on the fitting results of the initial volumetric creep a_2_ and confining pressure *σ*_3_ in Fig. 21, the relationship between other parameters c_5_ and stress level L, as shown in Fig. 22. The relationship between c_5_ and stress level L follows a quadratic function; therefore, it is expressed by a quadratic function. Consequently, the relationship between c_5_ and stress level L can be formulated as:

9 $${c}_{5}=0.091+0.389L-0.302{L}^{2}$$

Substituting Eq. (9) into Eq. (8) gives the initial volumetric creep a can be expressed as:

10 $${a}_{2}=1-{{\sigma }_{3}}^{0.091+0.389L-0.302{L}^{2}}$$

#### Volumetric creep exponent b

**Figure 23:**
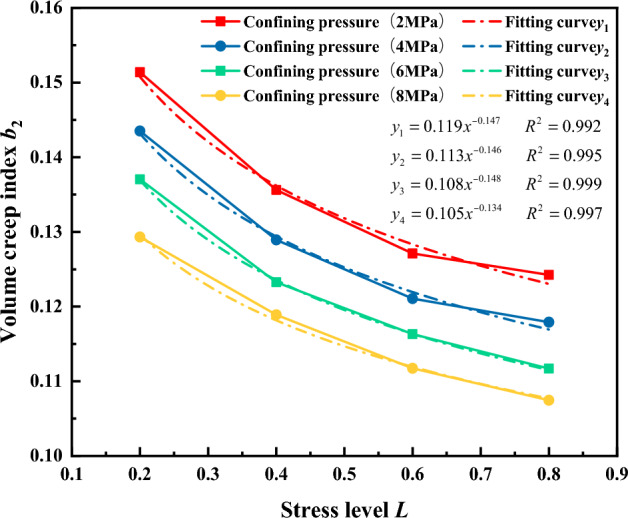
The relationship between the volumetric creep exponent b_2_ and the stress level.

**Figure 24:**
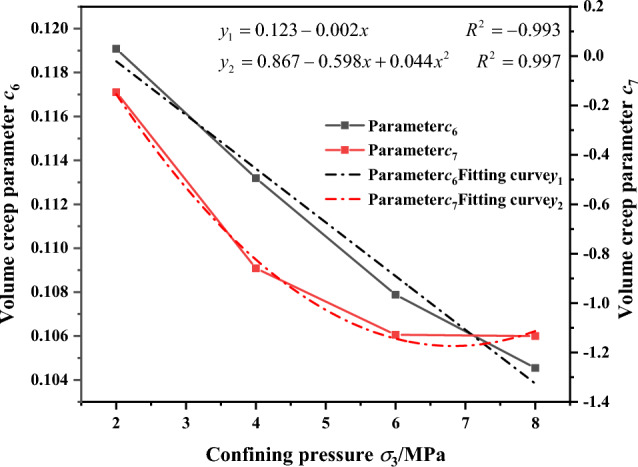
Relationship between volumetric creep parameters and confining pressure.

From the analysis it is considered that the stress level is the primary factor influencing the volumetric creep exponent, with the confining pressure being a secondary influencing factor. Therefore, when determining the expression for the volumetric creep exponent, the stress level is considered first, followed by the influence of the confining pressure. Based on the results of the logarithmic relationship in Fig. 24 and the volumetric creep-related parameters in Table 4, the relationships between the volumetric creep exponent *b*_2_ and the stress level L and confining pressure *σ*_3_ were fitted individually. The fitting results are shown in Figs. 23 and 24. It can be observed in Fig. 23 that the relationship between the volumetric creep exponent b_2_ and the stress level L indicates a gradual decrease in the axial initial creep of the gangue material with increasing stress level. Similarly, the relationship between initial axial creep and stress level can be described by the power function ${b}_{2}={c}_{6}{L}^{{c}_{7}}$. Based on the results of the volumetric creep exponent b_2_ and stress level L in Fig. 23, the relationships among other volumetric creep parameters c_6_, c_7_ and confining pressure *σ*_3_ were established, as illustrated in Fig. 24. As shown in Fig. 24, the relationships between *c*_6_, *c*_7_, and the confining pressure *σ*_3_ follow linear and quadratic functions and are expressed using linear and quadratic functions respectively. This gives the relationships between a, b and the confining pressure c:

11 $$\begin{array}{c}{c}_{6}=0.123-0.002{\sigma }_{3}\\ {c}_{7}=0.866-0.597{\sigma }_{3}+0.043{{\sigma }_{3}}^{2}\end{array}$$

Substituting c_6_ and c_7_ into the power function ${b}_{2}={c}_{6}{L}^{{c}_{7}}$, the initial volumetric creep b_2_ can be expressed as follows:

12 $${b}_{2}=\left(0.123-0.002\right){\sigma }_{3}{L}^{0.866-0.597{\sigma }_{3}+0.043{\sigma }_{3}^{2}}$$

Substituting Eqs. (10) and (12) into Eq. (1), the expression for the volumetric creep power-law constitutive model is as follows

13 $${\varepsilon }_{rv}=\left(1-{{\sigma }_{3}}^{0.091+0.389L-0.302{L}^{2}}\right){\left(\frac{t}{180}\right)}^{\left(0.123-0.002\right){\sigma }_{3}{L}^{0.866-0.597{\sigma }_{3}+0.043{\sigma }_{3}^{2}}}$$

## Conclusion

In this study, the creep and deformation characteristics of gangue backfill material under triaxial compression conditions were investigated and a power-law constitutive model for the gangue backfill material was developed. The creep characteristics of the gangue backfill material were systematically discussed in terms of particle size, stress and confining pressure. According to the analysis of the experimental data, some principal conclusions are drawn as follows.

1. The developed large scale three-directional compression test system for gangue backfill materials. This consists of three components: a loading system for applying axial and horizontal stresses, a three-directional compression chamber for filling gangue backfill material, and a data monitoring and acquisition system for experimental recording. This system makes it possible to study the compressive properties of gangue under different confining pressures.
2. The magnitude of the creep deformation growth rate for gangue specimens is as follows: single grain size grading > discontinuous grading > continuous grading. The overall trend of the creep deformation growth rate for the gangue shows a decreasing pattern. At low confining pressures, gangue materials are affected by lateral deformation, resulting in volumetric creep deformation being greater than their axial creep deformation for different gradations.
3. Creep deformation of gangue materials occurs primarily at low stress levels. The volumetric creep of gangue specimens is generally less under low confining pressure conditions than under high confining pressure conditions. Conversely, axial creep is greater under low confining pressure conditions than under high confining pressure conditions. During the creep process of gangue, deformation is mainly manifested as volumetric compression. Lateral contraction of gangue specimens under confining pressure is a significant contributor to creep deformation.
4. The fine fragments produced by the creep deformation of the rock. The sliding and filling of these fractured particles into pores are the main causes of creep deformation. The particle fragmentation rate increases continuously during the creep process. Under the action of an external force, the movement, recombination, and particle fragmentation effects of the gangue particles collectively induce creep deformation.
5. Express the three-directional compression creep characteristics of gangue backfill materials using a power function. Determination of initial axial (or volumetric) creep a and creep exponent b from three-directional compression creep tests. The power-law constitutive models for axial and volumetric creep, established in double logarithmic coordinates, show excellent applicability.

## Data Availability

The datasets generated during or analysed during the current study are available from the corresponding author on reasonable request.
